# Filament assembly underpins the double-stranded DNA specificity of AIM2-like receptors

**DOI:** 10.1093/nar/gkad090

**Published:** 2023-03-02

**Authors:** Archit Garg, Christina M Stallings, Jungsan Sohn

**Affiliations:** Department of Biophysics and Biophysical Chemistry, Johns Hopkins University School of Medicine, Baltimore, MD 21205, USA; Department of Biophysics and Biophysical Chemistry, Johns Hopkins University School of Medicine, Baltimore, MD 21205, USA; Department of Biophysics and Biophysical Chemistry, Johns Hopkins University School of Medicine, Baltimore, MD 21205, USA; Department of Oncology, Johns Hopkins University School of Medicine, Baltimore, MD 21205, USA; Divisions of Rheumatology, Johns Hopkins University School of Medicine, Baltimore, MD 21205, USA

## Abstract

Upon sensing cytosolic- and/or viral double-stranded (ds)DNA, absent-in-melanoma-2 (AIM2)-like-receptors (ALRs) assemble into filamentous signaling platforms to initiate inflammatory responses. The versatile yet critical roles of ALRs in host innate defense are increasingly appreciated; however, the mechanisms by which AIM2 and its related IFI16 specifically recognize dsDNA over other nucleic acids remain poorly understood (i.e. single-stranded (ss)DNA, dsRNA, ssRNA and DNA:RNA hybrid). Here, we find that although AIM2 can interact with various nucleic acids, it preferentially binds to and assembles filaments faster on dsDNA in a duplex length-dependent manner. Moreover, AIM2 oligomers assembled on nucleic acids other than dsDNA not only display less ordered filamentous structures, but also fail to induce the polymerization of downstream ASC. Likewise, although showing broader nucleic acid selectivity than AIM2, IFI16 binds to and oligomerizes most readily on dsDNA in a duplex length-dependent manner. Nevertheless, IFI16 fails to form filaments on single-stranded nucleic acids and does not accelerate the polymerization of ASC regardless of bound nucleic acids. Together, we reveal that filament assembly is integral to nucleic acid distinction by ALRs.

## INTRODUCTION

Although a universal building block of life, unchromatinized double-stranded (ds)DNA emerging in various cellular compartments signals major calamities (1–5). For example, ionizing irradiation and toxic chemicals damage mitochondria and/or nuclear dsDNA, resulting in accumulation of naked endogenous dsDNA in the cytosol and nucleus. Moreover, replicating viruses and bacteria produce long contiguous foreign dsDNA ranges in kilo- to mega base-pairs (bp). The host innate immune system plays a central role in responding to such rogue dsDNA by initiating inflammatory responses (1–5).

**Figure 1.**
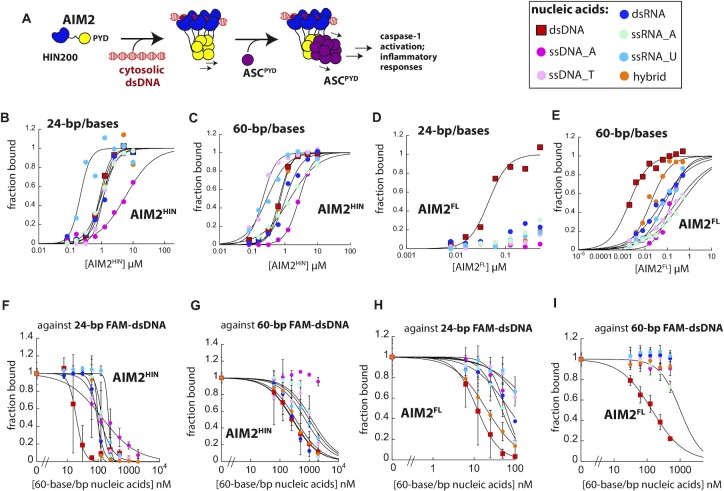
The preference for dsDNA increases with length. (**A**) A scheme describing the dsDNA binding, filament assembly, and signaling (polymerization of ASC) of AIM2. Only ASC^PYD^ is shown for simplicity (i.e. the CARD of ASC is omitted). Shown in B-E are representatives of three independent experiments. (**B, C**) Binding of AIM2^HIN^ toward various FAM-labeled nucleic acids (6 nM) was determined by FA. The lines are fits to the Hill form of binding isotherm. (**D, E**) Binding of AIM2^FL^ toward various FAM-labeled nucleic acids (3 nM) was determined by FA. The lines are fits to the Hill form of binding isotherm. (F, G) Competition binding assay using 24- or 60-bp FAM-dsDNA (6 nM) and AIM2^HIN^ (625 nM (**F**) and 400 nM (**G**)) against various 60-base/bp unlabeled nucleic acids; the lines are fits to a competition binding equation: (1/(1 + (competitor)/IC_50_)^Hill constant^). Fraction bound was calculated based on the changes FA of 60-bp FAM-dsDNA. Shown in (F)–(I) are averages of three experiments with error bars (standard deviation). (H, I) Competition binding assay using 24- or 60-bp FAM-dsDNA (6 nM) and AIM2^FL^ (100 nM (**H**) and 60 nM (**I**)) against various 60-base/bp unlabeled nucleic acids; the lines are fits to the competition binding equation. Fraction bound was calculated based on the changes FA of 60-bp FAM-dsDNA.

Absent-in-melanoma-2 (AIM2)-like receptors (ALRs) are a family of innate immune sensors that play vital yet versatile roles in defense against the emergence of unchromatinized dsDNA (3,6–13). For example, the eponymous member of the family, AIM2, assembles into filaments on cytosolic dsDNA and triggers the polymerization of ASC (apoptosis-associated speck forming protein containing caspase recruiting domain (CARD)), subsequently inducing the polymerization and activation of the caspase-1 protease (thus dubbed as the AIM2-ASC inflammasome) (6–8). Activated caspase-1 then executes pyroptotic cell death and proteolytic maturation of pro-inflammatory cytokines such as interleukin-1β (IL-1β) and interleukin-18 (IL-18) (Figure 1A) (6–8). AIM2 is crucial for host defense against various pathogens such as human papilloma virus (HPV), *F. novicida*, and even SARS-CoV2 (14–16). Moreover, it is integral to regulating dsDNA damage responses, tumor formation and growth and autoimmunity (17–22).

Interferon-inducible protein 16 (IFI16) is another major ALR that plays an important role in host defense against rogue dsDNA (1–5). Unlike AIM2, IFI16 is predominantly localized in the nucleus (9,12,23–26). Although seemingly counterintuitive for the host to localize a pro-inflammatory dsDNA sensor in the nucleus, IFI16 does not react to nuclear dsDNA as nucleosomes sterically block its polymerization necessary for signaling (12,23,24,27). Yet, IFI16 rapidly assembles into filaments on naked viral dsDNA once it breaches the nucleus, inducing inflammatory responses and restricting viral replication (e.g. Kaposi sarcoma herpes virus (KSHV) and herpes simplex virus (HSV) genomic dsDNA) (12,23,24). Moreover, the presence of cytosolic dsDNA exports IFI16, allowing it to form filaments and initiate inflammatory responses (9,23,25). Also unlike AIM2, IFI16 has diverse roles in innate immunity as it can promote either type-I interferon (IFN-I) or inflammasome (IL-1β and cell death) pathways depending on the nature of infected cells and pathogens (9,12,13,24,25,28). Overall, IFI16 is highly relevant to human health well beyond its role in defense against a wide variety of pathogens (9,12,13,24,25,28), as its dysregulation is associated with multiple autoimmune disorders (e.g. Sjögren's syndrome, systemic lupus erythematosus (SLE), and scleroderma (13,25,26,29)) and even cancer (e.g. breast, cervical and melanoma (30–33)).

Both AIM2 and IFI16 are well-established dsDNA sensors whose biological functions are increasingly more appreciated (1–5). However, the mechanisms that define their dsDNA specificity remain poorly understood at the molecular level. For instance, ALRs share the common domain organization in which the oligomerizing pyrin-domain (PYD) at the N-terminus is followed by one or two dsDNA-binding HIN200 domains (e.g. Figure 1A; HIN200: hematopoietic interferon-inducible nuclear antigen with a 200 amino-acid repeat). The HIN200 domains contain two oligonucleotide-binding (OB)-folds found in prokaryotic single-stranded (ss)DNA-binding proteins and interact with dsDNA exclusively on the phosphate backbone (34–36). Moreover, the HIN200 domains bind dsDNA transiently and filament assembly by distal PYD is necessary for stable binding (e.g. Figure 1A) (27,35–37); the PYD and HIN200 are separated by an unstructured linker composed of ∼50 and ∼100 amino acids for AIM2 and IFI16, respectively. Consequently, the length of dsDNA plays a major role in regulating the signaling activity of ALRs by dictating the probability (number of proximal ALRs) of assembling filaments necessary not only for stable binding, but also for interacting with downstream effectors (e.g. ASC for AIM2) (8,9,27,35–37). Such a dsDNA length-dependent activation mechanism provides an intuitive defense strategy (1–5,38), as longer duplexes would indicate major catastrophes (e.g. massive dsDNA damage and/or viral genome) while shorter fragments would signal minor repair or degraded pathogen genomes (i.e. resolution of such calamities). Nonetheless, currently, fundamental biological questions as to whether and how AIM2 and IFI16 are even capable of distinguishing dsDNA from other noncognate nucleic acids remain unanswered. Further confounding these questions, recent studies implicate IFI16 in defense against influenza viruses by directly detecting their RNA genome (39,40), indicating that its nucleic acid binding capabilities indeed reach beyond dsDNA.

We find here that the HIN200 domain of AIM2 does not show any preference for dsDNA (e.g. versus dsRNA, ssDNA, ssRNA and the DNA:RNA hybrid). However, full-length AIM2 binds to and oligomerizes on dsDNA most readily in a duplex length-dependent manner. Moreover, although AIM2 still assembles filaments on various nucleic acids other than dsDNA, these filaments not only appear less ordered, but also fail to accelerate the polymerization of downstream ASC. Similar to AIM2, the HIN200 domains of IFI16 do not strongly favor dsDNA, but the full-length protein preferentially binds to and oligomerizes on dsDNA most readily. Interestingly, we find that IFI16 has intrinsically higher affinity for RNA than AIM2, which in turn supports its role in detecting pathogenic RNA (39,40). Moreover, single-stranded nucleic acids fail to support filament assembly, and IFI16 does not directly accelerate the polymerization of ASC regardless of bound nucleic acids. Also of note, our experiments raise a new regulatory strategy for ALRs in which endogenous RNA would act as a buffer for attenuating spurious activations of ALRs against shorter dsDNA without interfering with their activation targeting pathogenic (long) dsDNA. Together, our investigation reveals that filament assembly is directly coupled to dsDNA-dependent activation and signaling of ALRs.

## MATERIALS AND METHODS

### Recombinant proteins

Human AIM2 full-length (AIM2^FL^, residues 1–343) and its HIN200 domain (AIM2^HIN^, residues 144–343) were cloned into the pET28b vector with an N-terminal His_6_-MBP tag and a tobacco etch virus protease (TEVp) cleavage site. Human IFI16^FL^ (residues 1–729) was cloned into the pET21b vector with a C-terminal His_6_-tag. The dsDNA binding domain of IFI16 (IFI16^HinAB^, residues 159–729) was cloned into the modified pET28b vector with an N-terminal His_6_-SUMO tag. All plasmids were transformed into *Escherichia coli* BL21-Rosetta 2^DE3^ cells and purified via Ni^2+^-NTA, cation exchange and size exclusion chromatography (storage buffer: 40 mM HEPES–NaOH at pH 7.4, 400 mM NaCl, 1 mM DTT, 1 mM EDTA and 10% glycerol). Proteins were then concentrated and stored at –80°C. See also (35,36).

### Nucleic acids

All oligonucleotides, nucleic acid mimics (low molecular weight variants), and yeast total RNA were purchased from Integrated DNA Technology (IDT), Invivogen, and ThermoFisher, respectively. Nucleic acid sequences are listed in Supplementary Table S1. 150-, 300- and 600-bp dsDNA fragments were generated via PCR using the maltose binding protein (MBP) sequence (35,36).

### Biochemical assays

Electrophoretic mobility shift assay (EMSA), fluorescence anisotropy (FA), and Förster resonance energy transfer (FRET)-based assays were conducted as described previously (35–37,41). MBP (for AIM2^FL^ and AIM2^HIN^) and SUMO (for IFI16^HinAB^) tags were cleaved by preincubation with either TEVp and Ulp1, respectively for 30 min before all measurements and imaging experiments. All biochemical assays with AIM2^FL^, AIM2^HIN^ and IFI16^FL^ were performed using the buffer containing 40 mM HEPES at pH 7.4, 160 mM KCl, 1 mM EDTA, 0.1% Triton X-100, 5 mM DTT and 5% glycerol. Assays with IFI16^HinAB^ were performed using the same buffer system with 60 mM KCl instead of 160 mM. For EMSA, the reaction mixture was incubated for 30 min then separated by 10% polyacrylamide gels (4°C for 75 min at 80 V). FRET and FA measurements were recorded using the Tecan M1000 or BioTek Synergy H1 plate reader at room temperature (23 ± 3°C). Kaleidagraph (Synergy Soft) was used to fit and analyze the data. Data shown are either representatives or averages of at least three independent experiments.

### Negative-stain electron microscopy (nsEM)

Each protein and nucleic acid complex sample was incubated for 30 min, applied to carbon-coated grids, and imaged using the Thermo-Fisher Talos L120C G2 transmission EM as described in (36,37,42).

## RESULTS

### The HIN200 domain of AIM2 binds various nucleic acids indiscriminately

Currently, if and how ALRs distinguish dsDNA from other intracellular nucleic acids remain unknown. To address this persisting fundamental mechanistic question in innate immunology, we fist tracked the changes in fluorescence anisotropy (FA) of fluorescein-amidite (FAM)-labeled nucleic acids upon binding recombinant AIM2 variants (the dsDNA-binding HIN200 domain in isolation (AIM2^HIN^) and the full-length (AIM2^FL^)). Considering that ALRs form filaments along the length of dsDNA (35–37), we decided to focus our investigations on determining whether these sensors can distinguish dsDNA from other linear nucleic acid types such as dsRNA, ssDNA, ssRNA and the DNA:RNA hybrid (Supplementary Table S1). We ensured that single-stranded species lack any secondary structures, and used two sequence variants for ssDNA and ssRNA to test any potential base•side-chain interactions (enriched with dA, dT, rA or rU; purine versus pyrimidine; Supplementary Table S1). Moreover, because dsDNA length is an important parameter for filament assembly and signaling by ALRs (8,9,27,35–37), we used ‘short’ (24 base/base-pairs (bps); suboptimal for filament assembly (35–37)) and ‘long’ (60-bases/bps; long enough to induce oligomerization (35–37)) nucleic acids.

In our FA assays testing 24-base/bp nucleic acids, there was no indication that AIM2^HIN^ specifically recognizes dsDNA (Figure 1B; Supplementary Table S2; see Supplementary Figure S1 for the data presented in Figure 1B–I plotted using changes (Δ) in FA). For instance, A-rich ssDNA showed the worst affinity and U-rich ssRNA bound most tightly (Figure 1B; Supplementary Table S2). Yet, AIM2^HIN^ bound dsDNA with essentially the same affinity as T-rich ssDNA, A-rich ssRNA, dsRNA and the hybrid (Figure 1B; Supplementary Table S2). 60-base/bp nucleic acids enhanced the binding affinity toward AIM2^HIN^ ∼2-fold compared to 24-base/bp fragments regardless of their identity; however, it still did not show any indication for dsDNA preference (Figure 1C, Supplementary Table S2). We also conducted electrophoretic mobility shift assays (EMSAs) using FAM-labeled nucleic acids to visually track the binding of AIM2^HIN^, which again showed no clear preference for dsDNA (Supplementary Figure 2A, B). These results indicate that the HIN200 domain of AIM2 does not chemically distinguish dsDNA from other nucleic acids.

### AIM2^FL^ preferentially binds dsDNA in a nucleic acid length-dependent manner

Although the HIN200 domains directly bind dsDNA, filament assembly by distal PYDs substantially enhances the overall dsDNA binding affinity of ALRs (35,36). Since our experiments suggested that nucleic acid distinction does not occur at the dsDNA binding domain of AIM2, we then tested whether oligomerization by the full-length protein has a role. Here, consistent with our previous report (36), AIM2^FL^ bound dsDNA at least 10-fold more tightly than AIM2^HIN^ (Figure 1B, C versus D, E and Supplementary Table S3). Notably, AIM2^FL^ favored 24-bp dsDNA over any other nucleic acids by at least 5-fold (Figure 1D, Supplementary Table S3). Moreover, although AIM2^FL^ bound all 60-base/bp nucleic acids more tightly than the shorter fragments, dsDNA was favored over other nucleic acid species by at least 10-fold (Figure 1E, Supplementary Table S3). EMSA results also corroborated that AIM2^FL^ binds 60-bp dsDNA most tightly (Supplementary Figure S2C, D). These results suggest that the dsDNA length-dependent filament assembly by AIM2 plays a major role in distinguishing its cognate ligand from other nucleic acids.

Rogue dsDNA would emerge in an intracellular environment where many other types of nucleic acids also exist in large abundance (e.g. mRNA and tRNA). We thus tested whether the length of duplex would still be a factor for selectively engaging dsDNA in the presence of other nucleic acids. Here, we used a competition method in which a fixed amount of AIM2^HIN^ or AIM2^FL^ was added last to reaction wells containing FAM-dsDNA and increasing concentrations of other unlabeled nucleic acids (i.e. unbiased competition) (35,36). For AIM2^HIN^, we found that all 60-base/bp nucleic acids competed well against both 24-bp and 60-bp FAM-dsDNA (Figure 1F, G, Supplementary Table S4), supporting the idea that the dsDNA-binding domain of AIM2 does not have any intrinsic preference. Of note, A-rich ssDNA failed to compete against 60-bp FAM-dsDNA, which is consistent with its poor affinity seen from our direct binding experiments (Figure 1G versus C). For AIM2^FL^, although dsDNA was most effective, various 60-base/bp nucleic acids were also able to compete against 24-bp FAM-dsDNA (Figure 1H, Supplementary Table S5). Strikingly, however, 60-bp FAM-dsDNA was significantly more favored over any other nucleic acids: only A-rich ssRNA marginally effective (∼10-fold weaker IC_50_ than dsDNA) and all other non-dsDNA species failed to compete against a sub-stoichiometric amount of 60-bp FAM-dsDNA (Figure 1I, Supplementary Table S5). Together, our observations consistently suggest that dsDNA length-dependent oligomerization underpins the nucleic acid distinction by AIM2.

### dsDNA provides the best platform for filament assembly and signaling

**Figure 2.**
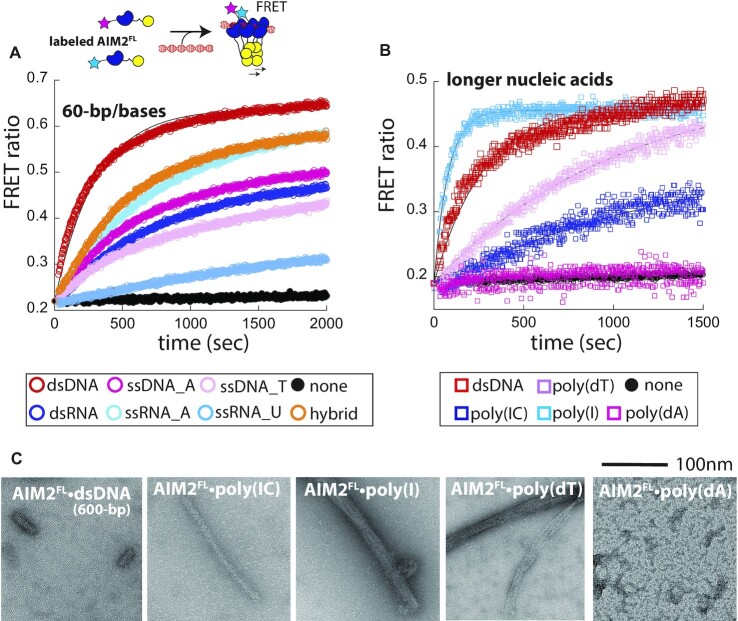
dsDNA provides the best platform for filament assembly. (**A**) Top: A cartoon describing the FRET-based polymerization assay. Only one pair of FRET-donor and acceptor is shown in the oligomer for simplicity. Bottom: the polymerization of FRET donor- and acceptor labeled AIM2^FL^ (37.5 nM each) on various 60-base/bp nucleic acids was monitored over time. Lines are fits to the single exponential growth equation. Shown in (A) and (B) are representatives of three separate experiments. (**B**) The time-dependent polymerization of FRET donor- and acceptor-labeled AIM2 (4.5 nM each) on 300-bp dsDNA and other nucleic acid mimics (>500 base/bp, nominal). Lines are fits to the single exponential growth equation. We used lower [AIM2^FL^] here than those used in the experiments with 60-base/bp fragments (A) to slow down the oligomerization and facile measurements (i.e. instrument deadtime). (**C**) nsEM images of AIM2^FL^ (250 nM) polymers assembled on various nucleic acids (10 ng/μl).

The assembly of ALR filaments is controlled kinetically (27,37). That is, once assembled, AIM2 and IFI16 filaments do not dissociate (i.e. no off-rate; (27,37)) and even persist indefinitely (25,26). dsDNA kinetically regulates the filament assembly of ALRs in a duplex length dependent manner, as longer duplexes accelerate the assembly by increasing the probability for forming the minimal oligomeric clusters (nucleation units) on the one-dimensional (1D) diffusion scaffold (27,37). Our results thus far consistently support the idea that filament assembly plays a major role in the dsDNA selectivity of AIM2. To further investigate this mechanism, we determined the oligomerization kinetics of AIM2^FL^ on various nucleic acids by tracking the Förster resonance energy transfer (FRET) ratios between acceptor- and donor-labeled proteins (Figure 2A top, see also (36,37,43)). Consistent with our binding experiments, dsDNA resulted in the fastest oligomerization of AIM2^FL^ amongst different 60-bp/base nucleic acids (Figure 2A, Supplementary Table S6; 24-bp dsDNA does not produce robust FRET signals (37)). We also tested the polymerization kinetics of AIM2^FL^ on much longer nucleic acids (mimics). Interestingly, a ssRNA-mimic (poly(I)) resulted in ∼3-fold faster polymerization kinetics than those observed from 300-bp dsDNA (i.e. optimal dsDNA length (37); Figure 2B, Supplementary Table S6). On the other hand, polymerization rates on a dsRNA-mimic (poly(I:C)) and a ssDNA-mimic (poly(dT)) were significantly slower, while another ssDNA mimic (poly(dA)) failed to generate robust FRET signals (Figure 2B, Supplementary Table S6).

Our FRET-based polymerization assay reports on the increased proximity of AIM2^FL^ molecules on nucleic acid scaffolds, but not on their architectures (36,37,43). Thus, to test whether AIM2^FL^ still assembles into filaments on nucleic acids other than dsDNA, we imaged resulting complexes via nsEM. AIM2^FL^ assembled into visually similar filaments on poly(IC) as those seen from 600-bp dsDNA (Figure 2C; ∼25 nm diameter, see also (36,37)). AIM2 filaments assembled on poly(I) and poly(dT) appeared less ordered and more bundled as if AIM2 oligomerized across multiple strands (Figure 2C; >25 nm wide). Poly(dA) did not result in filament assembly (Figure 2C), which is consistent with its failure to produce robust FRET signals (Figure 2B) and the poor binding affinity of A-rich ssDNA (Figure 1); the FRET signals from A-rich ssDNA suggest that AIM2^FL^ forms non-filamentous (smaller) oligomers (Figure 2A). Taken together, our observations consistently support the idea that dsDNA provides the best 1D-scaffold for filament assembly, which in turn defines the nucleic acid specificity of AIM2.

### dsDNA is the only nucleic acid that allows AIM2 oligomers to accelerate the polymerization of ASC

**Figure 3.**
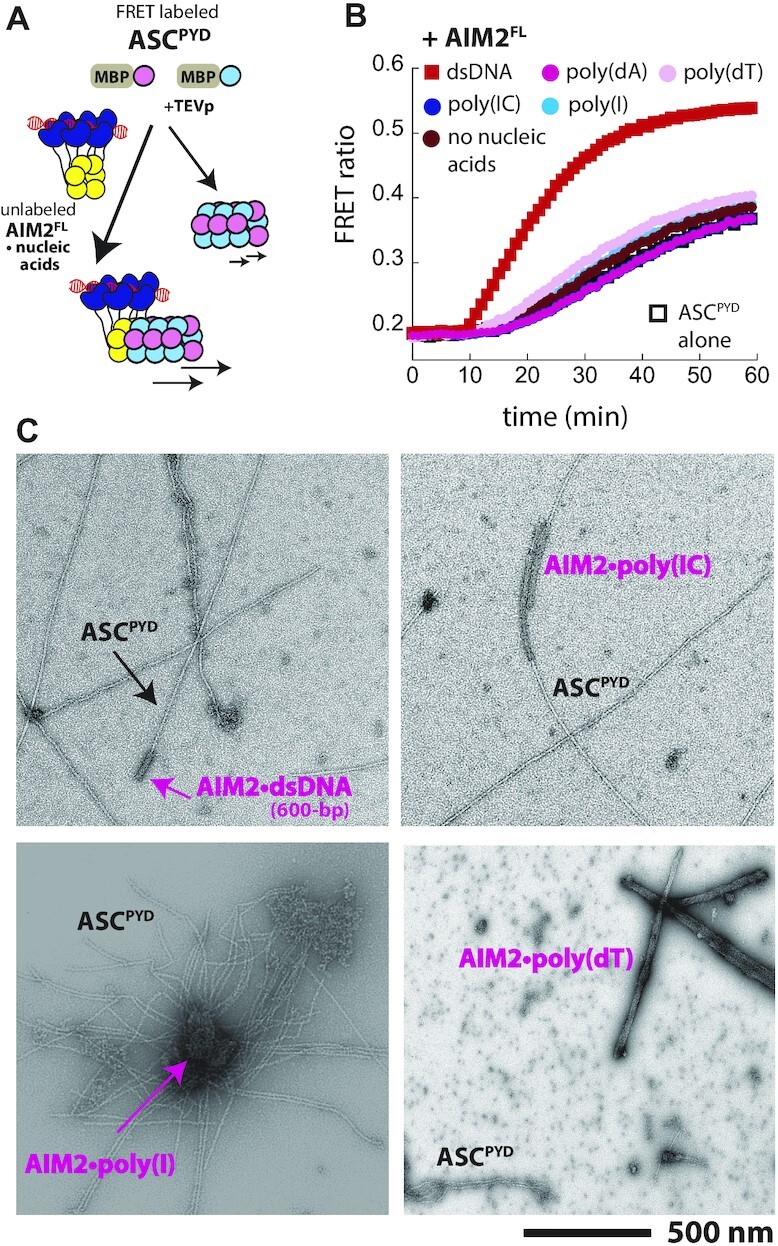
dsDNA is the only nucleic acid that allow AIM2 to signal through ASC. (**A**) A cartoon describing the FRET-based assay for tracking the polymerization of ASC^PYD^ in the presence and absence of AIM2 oligomers bound to various nucleic acids. (**B**) The time-dependent increase in FRET emission ratio of donor- and acceptor-labeled ASC^PYD^ (2.5 μM) was monitored in the presence of preassembled AIM2^FL^ (9 nM) on 10 ng/μl of indicated nucleic acids/mimics (preincubated for 30 min); note that total [AIM2] is the same as that used in Figure 2B. Shown is a representative of three independent experiments. (**C**) nsEM images of AIM2^FL^ bound to various nucleic acids (> 25 nm wide) and ASC^PYD^ filaments (∼9 nm wide). AIM2^FL^ (250 nM) was pre-incubated with each indicated nucleic acids/mimics (10 ng/μl) for 30 min; ASC^PYD^ (2.5 μM) was then added and further incubated for 30 min.

Our experiments here indicate that although AIM2 significantly prefers dsDNA, it can, in principle, bind to and assemble into filaments on various other nucleic acids (Figures 1 and 2). Nevertheless, the signaling activity of AIM2 via ASC has only seen from dsDNA *in vivo* (6–8), raising the question as to whether AIM2 filaments assembled on these other nucleic acids can interact with ASC. Moreover, our nsEM images suggested that AIM2 oligomers assembled on other nucleic acids, although filamentous, may not possess the same local architecture as AIM2•dsDNA filaments (Figure 2C). We thus tested whether AIM2^FL^ bound to various nucleic acids (mimics) can accelerate the polymerization of ASC^PYD^ to the same extents using our FRET-based polymerization assay (37,41,43). Briefly, the auto-assembly of the recombinant ASC^PYD^ can be suppressed by a N-terminal maltose binding protein (MBP) tag. Cleaving MBP via tobacco etch virus protease (TEVp) triggers filament assembly, which can be tracked via the FRET between donor- and acceptor-labeled ASC^PYD^; the presence of AIM2^FL^•dsDNA accelerates the polymerization of ASC^PYD^ in a dsDNA length-dependent manner (Figure 3A) (37,41,43). Here, as expected (37,41), AIM2^FL^•dsDNA robustly accelerated the polymerization of ASC^PYD^ (Figure 3B). By contrast, AIM2^FL^ bound to all other nucleic acids (mimics) only marginally enhanced the polymerization kinetics of ASC^PYD^ (Figure 3B), indicating that even if AIM2 polymerizes on nucleic acids other than dsDNA, it fails to signal through ASC. To further investigate the apparent lack of interactions between ASC^PYD^ and AIM2 polymers assembled on nucleic acids other than dsDNA, we imaged the upstream and downstream signaling partners together via nsEM. Here, we observed ASC^PYD^ filament (∼9 nm diameter; (44)) apparently growing off from one end of AIM2^FL^•dsDNA filaments (∼25 nm diameter; (36,37)) (Figure 3C), which is consistent with the notion that ASC assembles from one specific end of receptor filaments (37,41,45). Interestingly, ASC^PYD^ also appeared to make a directional contact with the AIM2^FL^•poly(IC) complexes (Figure 3C). Considering the lack of acceleration of ASC^PYD^ polymerization (Figure 3B), our observation suggests that AIM2^FL^•poly(IC) filaments likely contain altered local architectures suboptimal for inducing ASC^PYD^ assembly. On the other hand, AIM2•poly(I) oligomers appeared to be aggregated on top of ASC^PYD^ filaments (Figure 3C), and AIM2•poly(dT) filaments did not appear to interact with ASC^PYD^ filaments (Figure 3C). These observations suggest that AIM2 filaments assembled on nucleic acids other than dsDNA are not conducive to interacting with ASC^PYD^. Together, our experiments demonstrate that dsDNA provides the best signaling platform for AIM2 not only for initial assembly, but also for signal transduction through ASC.

### IFI16^FL^ preferentially assembles filaments on dsDNA, but shows broader nucleic acids specificity than AIM2

**Figure 4.**
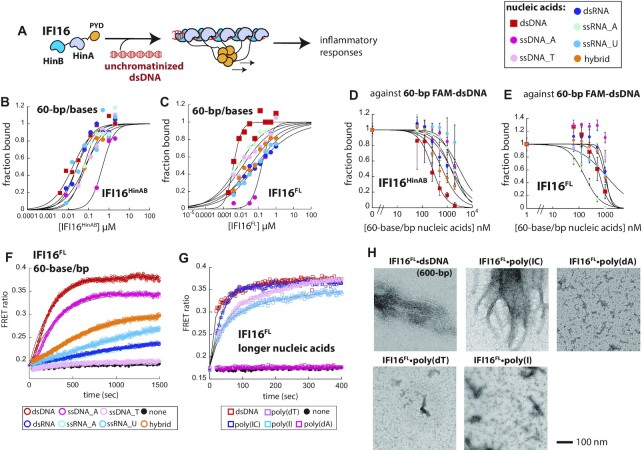
Nucleic acid binding properties of IFI16. (**A**) A scheme describing dsDNA binding, filament assembly, and signaling by IFI16. (**B, C**) Binding of IFI16^HinAB^ and IFI16^FL^ toward various 60-base/bp FAM-labeled nucleic acids (3 nM) was determined by FA. The lines are fits to the Hill form of binding isotherm. Shown are representatives of three separate experiments. (D, E) Competition binding assay using 60-bp FAM-dsDNA (6 nM) and IFI16^HinAB^ ((**D**), 400 nM) and IFI16^FL^ ((**E**), 100 nM) against various 60-base/bp unlabeled nucleic acids; the lines are fits to the competition binding equation. Fraction bound was calculated based on the changes FA of 60-bp FAM-dsDNA. Shown are averages of three experiments with error bars (standard deviation). (F, G) The polymerization of FRET donor- and acceptor-labeled IFI16^FL^ (150 nM each for (**F**) and 30 nM each for (**G**)) on 150-bp dsDNA and various other nucleic acids (mimics) was monitored over time. Data for (F) were fitted with a single exponential equation. Data for (G) was fitted using a variant the Hill equation to obtain the reaction half-time (*t*_1/2_): observed FRET ratio at time (*t*) = (max observed FRET ratio)/(1 + (*t*_1/2_/*t*)*^n^*), where *n* is an exponent that allows fitting to the lag-phase. The apparent assembly rates were then calculated by ln(2)/*t*_1/2_. See also (37). Shown are representatives of three separate experiments. (**H**) nsEM images of IFI16^FL^ (300 nM) oligomers assembled on various nucleic acids (10 ng/μl).

Although belonging to the same family, IFI16 diverges from AIM2 in two major ways. First, IFI16 has two HIN200 domains (Figure 4A) (9,34,35). Second, although AIM2 has been strictly implicated in dsDNA sensing (6–8,10), it was recently identified that IFI16 detects pathogenic RNA in addition (39,40). Thus, we next investigated whether and how IFI16 might be similar or different from AIM2 in coupling its filament assembly to nucleic acid selectivity. Of note, the HIN200 domains of IFI16 in isolation (IFI16^HinAB^) has intrinsically weak dsDNA binding activity that can only be studied under lower-than-physiological buffer ionic strengths (e.g. 60 mM KCl for IFI16^HinAB^ versus 160 mM for others) (27,35). We also noted that binding of IFI16^HinAB^ or IFI16^FL^ did not generate reliable FA signals other than dsDNA for 24-base/bp oligonucleotides. Thus, we first tracked the binding of IFI16^HinAB^ toward the shorter nucleic acids using EMSA and the low salt buffer. Here, we found that IFI16^HinAB^ does not favor one particular type of nucleic acid (Supplementary Figure S3A). For instance, for 24-base/bp fragments, dsDNA and the DNA:RNA hybrid appeared to bind most tightly with similar affinities (apparent *K*_D_ ≤ 250 nM), ssRNA fragments were favored in second (apparent *K*_D_ ∼ 500 nM), then dsRNA and ssDNA species bound most poorly (apparent *K*_D_ ≥ 1 μM; Supplementary Figure S3A). Moreover, all 60-base/bp nucleic acids bound to IFI16^HinAB^ with similar affinity (differences in *K*_D_ ≤ 3-fold), except for A-rich ssDNA, which bound ∼20-fold more weakly (Figure 4B, Supplementary Table S7); EMSA results agreed with our solution FA-based measurements (Supplementary Figure S3B). IFI16^FL^ appeared to disfavor ssDNA species in our EMSAs with 24-base/bp fragments, and failed show clear preference toward dsDNA (Supplementary Figure S3C). However, as seen from AIM2, IFI16^FL^ bound 60-bp dsDNA most tightly (Figure 4C, Supplementary Figure S3D, and Supplementary Table S8). We also noted that the fitted Hill constant was significantly higher for IFI16^FL^ binding 60-bp dsDNA (∼2) than other nucleic acids in the same length (≤1), indicating that only the DNA duplex allows cooperative oligomerization (Figure 4C, Supplementary Table S8; see below for A-rich ssDNA) (35); we refrained from drawing any conclusions on cooperativity for AIM2, as the fitted Hill constants did not show any strong correlations (Supplementary Table S2). Overall, these observations suggest that, as with AIM2, dsDNA-dependent oligomerization underpins the nucleic acid selection of IFI16.

There was no clear preference for any nucleic acids in competition against 24-bp FAM-dsDNA for IFI16^HinAB^ (Supplementary Figure S4A, Supplementary Table S9), but duplexes containing the DNA moiety (dsDNA and DNA:RNA hybrid) appeared to be more effective in competing against 60-bp FAM-dsDNA (Figure 4D, Supplementary Table S9). Nevertheless, in contrast to AIM2^FL^ that clearly favored dsDNA (Figure 1H, I), IFI16^FL^ appeared only to disfavor ssDNA in our competition binding experiments (Figure 4E, Supplementary Figure S4B, and Supplementary Table S10). For example, RNA species competed against FAM-dsDNA as effectively as dsDNA (or even better for A-rich ssRNA), but both A- and T-rich ssDNA showed minimal competition (Figure 4E, Supplementary Figure S4B, and Supplementary Table S10). These results indicate that although IFI16 preferentially recognizes dsDNA via its oligomerization-coupled binding mechanism, it has broader nucleic acid binding properties than AIM2.

We next determined the oligomerization kinetics of IFI16^FL^ on various nucleic acids by tracking the FRET ratio between donor- and acceptor-labeled proteins (27,35). Akin to AIM2^FL^, IFI16^FL^ oligomerized fastest on dsDNA for both 60-bp/base and the longer species (Figure 4F, G, Supplementary Table S11). When visualized using nsEM, dsDNA and poly(IC) produced similar IFI16^FL^ filaments; however, it appeared that the single-stranded species failed to support filament assembly (Figure 4H). Since poly(dT) and poly(I) produced robust FRET signals (Figure 4G), our observations indicate that IFI16 form non-filamentous oligomers (or bundled/aggregated) on these nucleic acids. Also of note, although A-rich ssDNA showed the worst affinity in our EMSA and FA-based binding assays, its binding appeared to be cooperative for both IFI16^HinAB^ and IFI16^FL^ (Hill constant ∼1.5; Figure 4B–E, Supplementary Figure S4A, B, Supplementary Tables 7 and 8). Moreover, 60-base A-rich ssDNA showed the second fastest oligomerization kinetics (Figure 4F), yet poly(dA) failed to produce robust FRET signals and filaments (Figure 4G, H). These results suggest that IFI16^FL^ likely forms non-filamentous (likely non-functional or alternate function) oligomers on A-rich ssDNA. Overall, our experiments consistently support the idea that filament assembly is tightly coupled to the dsDNA selectivity of IFI16.

Although it is well-established that AIM2 forms an inflammasome with ASC (10,11,37,41), there are conflicting views for IFI16. For example, although it was fist found that AIM2 is the only ALR that assembles into an inflammasome with ASC in response to cytosolic dsDNA (6–8,46), other studies found that IFI16 forms an inflammasome with ASC upon infection by certain viruses both in the nucleus and the cytosol (e.g. HIV and KSHV) (12,28). Considering that all prior conclusions are based on indirect measurements using cellular systems that harbor multiple inflammasome regulators from both hosts and pathogens (6–8,12,28), we tested whether recombinant IFI16^FL^ can accelerate the polymerization of ASC^PYD^. Here, we found that the presence of IFI16 failed to modulate the polymerization kinetics of FRET-labeled ASC^PYD^ regardless of the identity of bound nucleic acids (Supplementary Figure S4C), suggesting that IFI16 is unlikely to interact with ASC directly.

### Intracellular RNA could suppress spurious activation of ALRs

Our experiments raise three new mechanistic concepts in innate immune sensing of cytosolic dsDNA: First, ALRs selectively engage dsDNA over any other nucleic acids, as the former provides the best oligomerization platform for kinetically regulating their signaling pathways. Second, endogenous RNA could accentuate dsDNA length dependent activation by selectively competing against shorter dsDNA. Finally, even if ALRs (AIM2 in particular) assemble into nucleic acids other than dsDNA they would fail to signal through ASC. To further explore the physiological relevance of these ideas, we then investigated how ALRs would interact with a mixture of cytosolic RNA (yeast total RNA extract, YTR). For AIM2^HIN^, at least 5-fold more YTR was required than linear dsDNA fragments to compete against FAM-labeled species (Supplementary Figure S5A, Supplementary Table S12). Moreover, YTR competed against 24-bp FAM-dsDNA nearly 5-fold better than against 60-bp FAM-dsDNA (Supplementary Figure S5A, Table S12).

**Figure 5.**
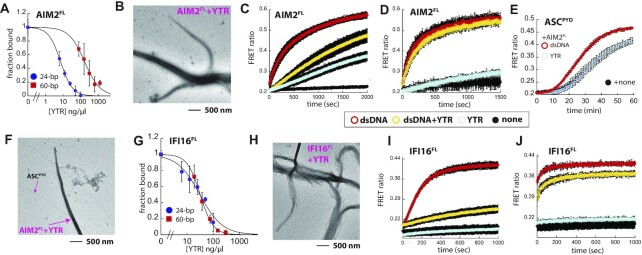
Cellular RNA could regulate the dsDNA sensing activity of ALRs. (**A**) Competition binding assays using 24- or 60-bp FAM-dsDNA (6 nM) and AIM2^FL^ (100 and 60 nM, respectively) against yeast total RNA extract (YTR). Shown are averages of three experiments with error bars (standard deviation). (**B**) A nsEM image of AIM2^FL^ (250 nM) oligomers assembled on YTR (300 ng/μl). (**C**) The polymerization of FRET donor- and acceptor-labeled AIM2^FL^ (37.5 nM each) against 60-bp dsDNA (10 ng/μl), YTR (600 ng/μl). Shown are averages of three experiments with error bars (standard deviation). (**D**) The polymerization of FRET donor- and acceptor-labeled AIM2^FL^ (4.5 nM each) against 300-bp dsDNA (10 ng/μl), YTR (600 ng/μl). Shown are averages of three experiments with error bars (standard deviation). (**E**) The time-dependent increase in FRET emission ratio of donor- and acceptor-labeled ASC^PYD^ (2.5 μM) was monitored in the presence of preassembled AIM2^FL^ (9 nM) on 10 ng/μl of 300 dsDNA or 600 ng/μl of YTR (preincubated for 30 min). Shown are averages of three experiments with error bars (standard deviation). (**F**) A nsEM image of AIM2^FL^ oligomers assembled on YTR (600 ng/μl) and ASC^PYD^ filaments. AIM2^FL^ (250 nM) was pre-incubated with YTR (600 ng/μl) for 30 min; ASC^PYD^ (2.5 μM) was then added and further incubated for 30 min. (**G**) Competition binding assays using 24- or 60-bp FAM-dsDNA (6 nM) and IFI16^FL^ (500 and 100 nM, respectively) against YTR. Shown are averages of three experiments with error bars (standard deviation). (**H**) A nsEM image of IFI16^FL^ (1 μM) oligomers assembled on YTR (300 ng/μl). (**I**) The polymerization of FRET donor- and acceptor-labeled IFI16^FL^ (150 nM each) against 60-bp dsDNA (10 ng/μl), YTR (300 ng/μl). Shown are averages of three experiments with error bars (standard deviation). (**J**) The polymerization of FRET donor- and acceptor-labeled IFI16^FL^ (30 nM each) against 150-bp dsDNA (10 ng/μl), YTR (300 ng/μl). Shown are averages of three experiments with error bars (standard deviation).

Strikingly, for AIM2^FL^, at least 20-fold more YTR was required to compete against FAM-dsDNA compared to linear dsDNA fragments. Moreover, the preference for the longer dsDNA further increased, as ∼25-fold more YTR was required to compete against 60-bp FAM-dsDNA than the 24-bp FAM-labeled fragment (Figure 5A, Supplementary Table S12). Visualizing YTR•AIM2^FL^ complexes using nsEM revealed that AIM2^FL^ still forms filamentous oligomers (Figure 5B). However, compared to dsDNA, significantly more YTR was required to induce the polymerization of AIM2^FL^ (60-fold more than linear dsDNA fragments; Figure 5C-D), the oligomerization kinetics were ignorantly slower (Figure 5C-D, red vs. cyan), and the changes in FRET amplitude were also substantially lower especially compared to 300-bp dsDNA (Figure 5D, red versus cyan). Additionally, AIM2^FL^ oligomers assembled on YTR failed to accelerate the polymerization of ASC^PYD^ (Figure 5E); no clear connections between AIM2^FL^ filaments and ASC^PYD^ filaments were observed in nsEM (Figure 5F). Because YTR-mediated FRET signals from AIM2^FL^ were much lower in amplitude and kinetics (Figure 5C, D, red versus cyan), we then tested whether YTR can interfere with the dsDNA-induced polymerization of AIM2^FL^. Here, the presence of a near-saturating concentration of YTR (4-times IC_50_, 60-fold higher than dsDNA in mass concentration) effectively suppressed the oligomerization kinetics of AIM2 on 60-bp dsDNA (Figure 5C, red versus yellow, ∼5-fold slower half-time). By contrast, the presence of YTR did not alter the polymerization kinetics on 300-bp dsDNA (Figure 5D, red versus yellow).

For IFI16^HinAB^, at least 8-fold more YTR was required than linear dsDNA to compete against FAM-labeled fragments (Supplementary Figure S5B; Supplementary Table S12); however, there was no clear preference for the longer FAM-dsDNA (Supplementary Figure S5B). Furthermore, YTR competed for IFI16^FL^ nearly as well as linear dsDNA, again without length dependence (Figure 5G; Supplementary Table S12). Nevertheless, significantly more IFI16 and YTR were required than dsDNA to assemble into filaments (3-fold and 30-fold, respectively; Figure 5H and Supplementary Figure S5C). In our oligomerization assay, YTR essentially failed to induce robust FRET signals (Figure 5I and J, red versus cyan). We then tested whether YTR can compete against dsDNA and found that the assembly on 60-bp dsDNA was significantly impacted, while the presence of YTR minimally affected the oligomerization on 150-bp dsDNA (Figure 5I, J, red versus yellow). Taken together, our results consistently support the idea that cellular RNA would suppress spurious activation of ALRs only against shorter dsDNA (minor repair/degraded pathogen genome) without interfering with their ability to engage long dsDNA arising from major catastrophes (e.g. intact viral genome and massive UV damage).

## DISCUSSION

AIM2 and IFI16 were identified in late 1990s as an IFN-inducible tumor suppressor and an autoantigen in SLE, respectively (47). Their biological functions had remained rather underappreciated for more than a decade, which changed dramatically in 2009 when AIM2 was identified as the cytosolic dsDNA receptor for activating ASC-dependent inflammasomes (6–8). Soon after, IFI16 was identified to be an important modulator for activating IFN-I responses against cytosolic dsDNA (9); IFI16 was also subsequently implicated in assembling into an inflammasome with ASC in response to certain viruses such as KSHV and HIV (12,28). The important roles of these two ALRs in host defense and human health are still increasingly more appreciated, which include regulating autoimmunity, tumorigenesis, atherosclerosis, and even normal neuronal development (1–3,10,11,13,22,38). Multiple biochemical and structural studies have also followed, providing key molecular insights into their activation and signaling mechanisms (27,34–37,41). Nevertheless, the very fundamental question as to how ALRs specifically recognize and signal through dsDNA has remain unanswered. Our study here reveals that filament assembly plays a major role in dsDNA selectivity of ALRs.

We and others have previously found that the length of dsDNA plays a major role in regulating the assembly and signaling activities of both AIM2 and IFI16 (8,9,35,36). The length of contiguous dsDNA is strongly correlated with the severity of intracellular maladies (1–5,38). For instance, long naked dsDNA well over kilo-bps would result from massive dsDNA damage or replicating pathogens, while much shorter dsDNA would indicate minor repairs and/or degraded viral genomes. Although their binding footprint is less than 15-bp, ALRs require at least 60-bp dsDNA to stably bind and initiate their signaling cascades both *in vitro* and in cells, and their optimal activities often require ≥150-bp contiguous dsDNA (8,9,35,36). This is because the HIN200 domains have intrinsically weak dsDNA binding affinity and the oligomerization (filament assembly) by PYDs leads to stable binding (27,35–37). Nevertheless, the transient interactions between the HIN200 domains and dsDNA allow ALRs to 1D diffuse on the duplex, allowing ALRs to find one another and initiate polymerization (27,37). Consequently, longer dsDNA not only leads to tighter binding, but also promotes faster oligomerization for ALRs (27,35–37).

**Figure 6.**
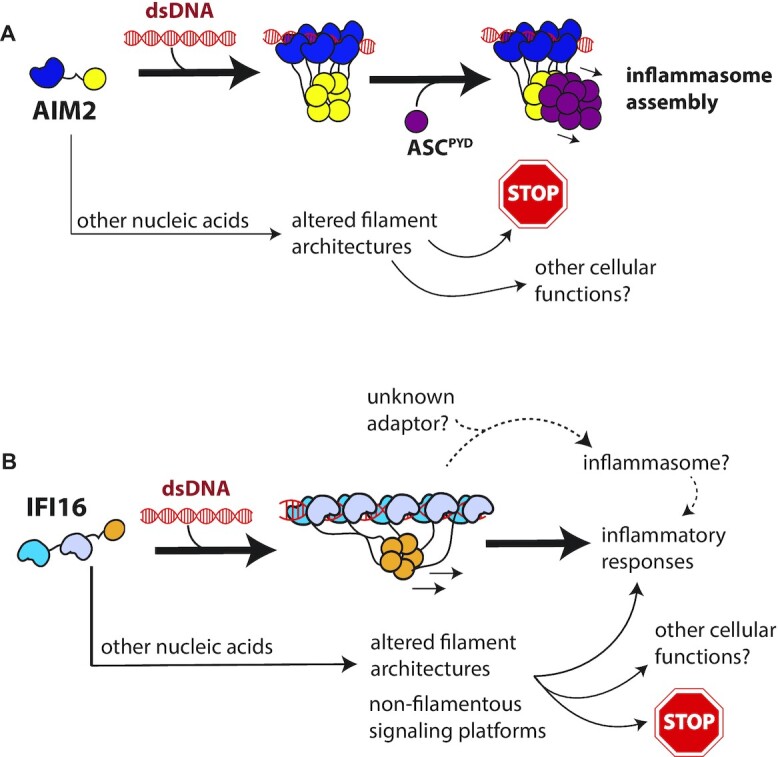
Nucleic acid distinction mechanisms of ALRs. (**A**) AIM2 assembles into filaments most readily (fastest) on dsDNA. AIM2 binds to and oligomerizes on other nucleic acids with weaker affinity (slower). Moreover, AIM2 filaments assembled on other nucleic acids would result in altered local and/or global structures not conducive to interacting with downstream ASC. (**B**) As with AIM2, IFI16 binds to and oligomerizes most readily on dsDNA. Single-stranded nucleic acids do not support filament assembly, which might lead to its altered cellular functions. Additionally, IFI16 filaments likely require yet unidentified adaptor to directly interact with ASC.

It is particularly noteworthy that our results raise a new mechanistic concept that cellular RNA can attenuate spurious activation of ALRs (AIM2 in particular) by selectively competing against their assembly on shorter dsDNA (Figure 5). Prior studies and our observations here consistently demonstrate that the HIN200 domain of ALRs do not display any preference for a particular type of nucleic acids ((7–9,34–36); e.g. Figures 1B and 4B), indicating that there are no specific side-chain-to-base interactions for dictating their ligand selectivity. However, not only do full-length proteins bind more tightly to, but also oligomerizes faster on dsDNA than any other nucleic acids in a duplex-length dependent manner (e.g. Figures 1E, 2A, 4C, F and 5D and J). Each nucleic acid type has unique physical properties such as width, persistence length, stiffness, and flexibility (48–50). It is thus tempting to speculate that single-stranded species would be too flexible to support the assembly of highly ordered structures such as filaments compared to dsDNA. Indeed, ALR oligomers assembled on single-stranded nucleic acids appear either non-filamentous or significantly more irregular than those assembled on double-stranded species (Figures 2C, 4H, 5B/H). On the other hand, it is likely that assembling filaments on dsRNA is not as conducive as that on dsDNA due to the higher stiffness of the ribonucleic acids (48–50). Furthermore, oligomerization on structured RNAs would be further hampered as evidenced by the lack of robust oligomerization activities on YTR (Figure 5). Overall, our experiments consistently indicate that dsDNA provides the best nucleic acid scaffold for ALRs to assemble signaling-competent filaments, thereby conferring their nucleic acid specificity (Figure 6A, B).

Once assembled into filaments, AIM2 induces the polymerization of ASC^PYD^ (6–8). Of note, AIM2^PYD^ and ASC^PYD^ filaments share the same helical architecture (36,41,44), which is consistent with the idea that the congruent supra-structures between the upstream and downstream filaments underpin their recognition (36,38,51–53). We find here that although AIM2 can assemble into filamentous oligomers on various nucleic acids, these polymers are virtually incapable of accelerating the polymerization of ASC^PYD^ (Figures 2, 3 and 5). Our observations suggest that nucleic acid distinction by AIM2 follows the dual-filter strategy seen from its dsDNA length dependent signaling mechanism (37). That is, we found previously that longer dsDNAs not only accelerate initial filament assembly, but also enhance the ability of AIM2 to induce the polymerization of ASC^PYD^, as the duplex length dictates the probability of forming the filament base necessary for recognition (37). Our quantitative model then revealed that the dependence on dsDNA length for the second signaling step is integral to preventing shorter dsDNA from activating the AIM2 pathway (37). Here, we find that although dsDNA is much preferred, AIM2 can assemble into filaments on various other nucleic acids within minutes (Figure 2, Supplementary Table S6). Nevertheless, these non-dsDNA AIM2 nucleoprotein filaments are essentially incapable of either interacting with or accelerating the polymerization of ASC^PYD^ (Figure 3). Although future structural studies will provide deeper insights into the exact mechanism, based on the lack of (productive) interaction with the ASC^PYD^, we speculate that AIM2 filaments assembled on nucleic acids other than dsDNA likely contain different local architectures from the congruent helical structures necessary to recognize the downstream adaptor (Figure 6A).

Similar to AIM2, our results here indicate that dsDNA-dependent filament assembly largely defines the nucleic acid selectivity of IFI16 (Figure 6B), revealing the unifying theme in dsDNA recognition by ALRs. However, compared to AIM2, IFI16 is more prone to binding various other nucleic acids (Figures 4E and 5G). We also noted that IFI16 does not assemble into filaments on single-stranded nucleic acids (Figure 4H). Considering its involvement in sensing non-dsDNA such as the RNA genome of influenza viruses (39,40), it is tempting to speculate that various binding modes and oligomeric states of IFI16 might steer its intracellular function (Figure 6B). Additionally, there has been conflicting reports on whether IFI16 forms an inflammasome. Indeed, unlike AIM2 (3,6–8,11,14–16,18–20,46), IFI16 inflammasomes have been implicated in very limited cases involving certain viruses (12,28). Our experiments using recombinant proteins strongly suggest that IFI16 does not directly induce the polymerization of ASC^PYD^ regardless of bound nucleic acids (Supplementary Figures 4C and 5D). It is possible that IFI16 filaments have intrinsically different helical architectures than those from AIM2 and/or ASC, precluding their direct interactions (the structure of IFI16^PYD^ filament remains unknown). Nevertheless, considering that the IFI16 inflammasome has only been sparsely observed (12,28), we speculate that a yet-unidentified host or pathogen proteins and/or post-translational modifications could provide the bridge between IFI16 and ASC for inflammasome assembly (Figure 6B).

A very basic question in innate immunology is how to distinguish self vs. non-self (54–56). For instance, unlike the adaptive system, the innate immune system detects certain molecular patterns associated with pathogenic conditions. Such patterns are sometimes unique to pathogens (e.g. bacterial lipo-polysaccharides and muramyl dipeptide) (54–56); however, nucleic acids, although a major danger signal, are universal to both hosts and pathogens (54–56). The emergence of naked dsDNA in the cytosol or nucleus is caused by major calamities such as pathogen invasion or exposure to ionizing irradiation (55,56). Because naked contiguous dsDNA is rare in either compartment, its conditional appearance can clearly mark such a ubiquitous molecule as a danger signal (55,56). Moreover, we and others have found that the length of dsDNA plays a major role for both AIM2 and IFI16 to gauge the severity of intracellular maladies, as duplex length regulates their oligomerization activity (8,9,35,36). Considering that ALRs do not display any sequence specificity (8,9,34–36), another glaring problem has been how to selectively recognize dsDNA when there is a plethora of other nucleic acids. Our study here revealed that filament assembly is integral to nucleic acid distinction by the ALR innate immune sensors (Figure 6A-B).

## DATA AVAILABILITY

All data will be available upon request.

## Supplementary Material

gkad090_Supplemental_File
