# Supplementary material for: Filament assembly underpins the double-stranded DNA specificity of AIM2-like receptors
Source: Nucleic Acids Res. 2023 Mar 2;51(6):2574–85. doi: 10.1093/nar/gkad090 (PMC10085679; doi:10.1093/nar/gkad090)
Supplement: gkad090_Supplemental_File [file gkad090_supplemental_file.pdf]

Supplementary Figure 1

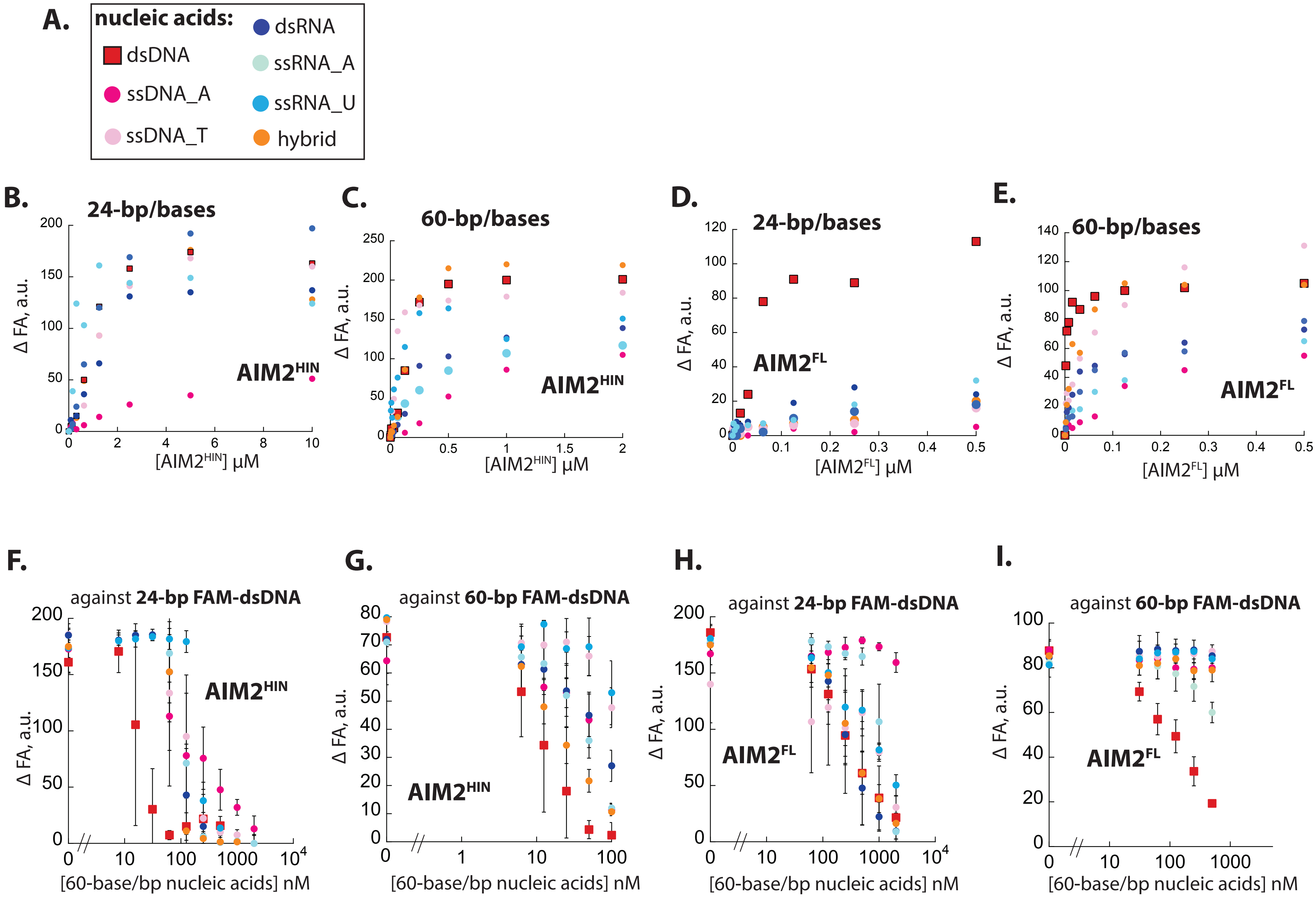

Supplementary Figure 1.

(**A**) Legends for B-I

(**B-I)** The same data sets from **Figure 1B-I** are plotted as changes (Δ) in raw FA values.

Supplementary Figure 2

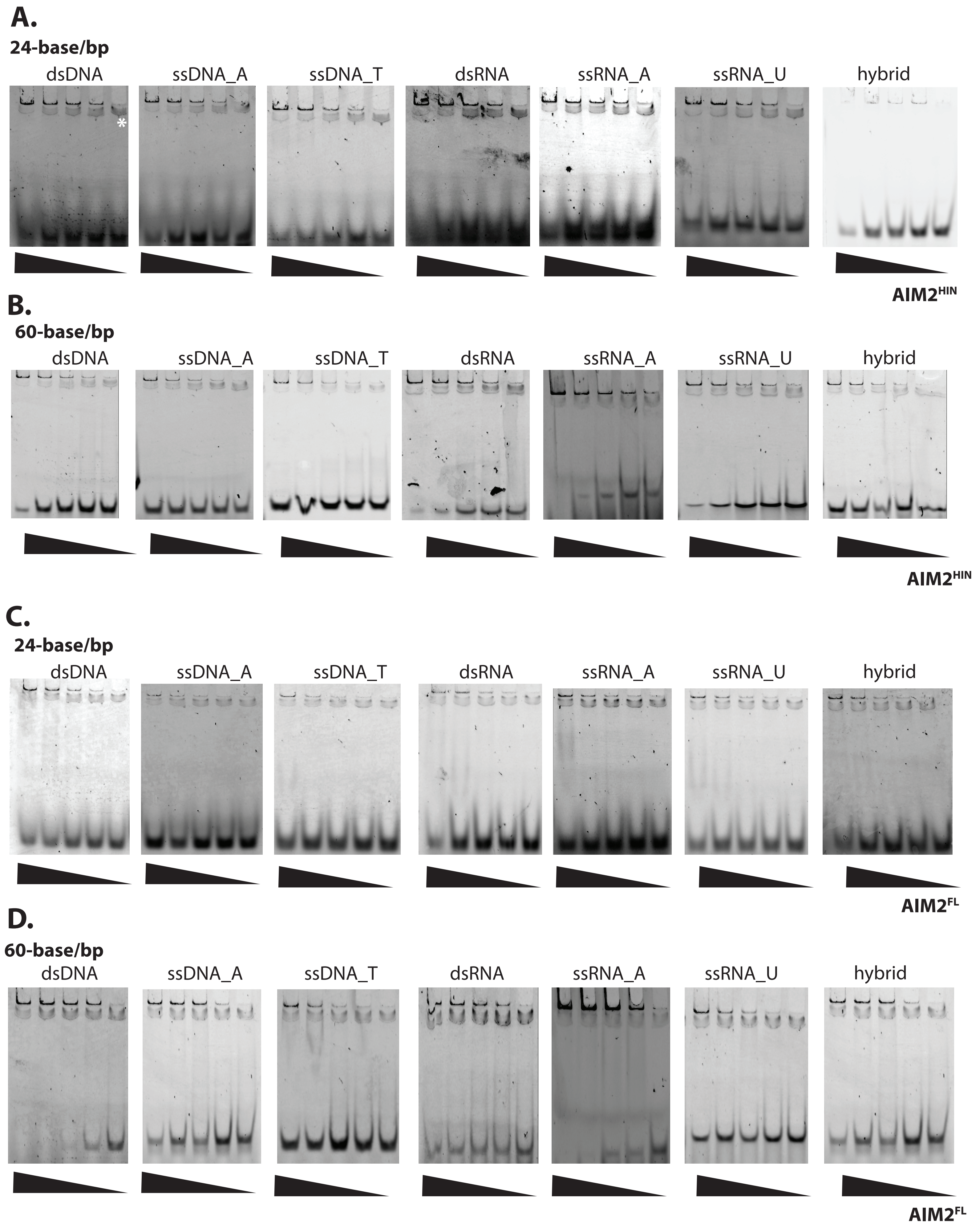

Supplementary Figure 2.

(**A**) EMSAs of $AIM2^{HIN}$ (from left to right: 5, 2.5, 1.25, 0.625, and 0 μM) binding various 24-bp/base FAM-labeled nucleic acids (20 nM). *: triton X-100 in the reaction buffer appears in florescence scanning (Typhoon, Cytiva).

(**B**) EMSAs of $AIM2^{HIN}$ (2, 1, 0.5, 0.25, and 0 μM) binding various 60-bp/base FAM-labeled nucleic acids (20 nM).

(**C**) EMSAs of $AIM2^{FL}$ (1, 0.5, 0.25, 0.125 and 0 μM) binding various 24-bp/base FAM-labeled nucleic acids (20 nM).

(**D**) EMSAs of $AIM2^{FL}$ (0.5, 0.25, 0.125, 0.0625 and 0 μM) binding various 60-bp/base FAM-labeled nucleic acids (20 nM).

Supplementary Figure 3

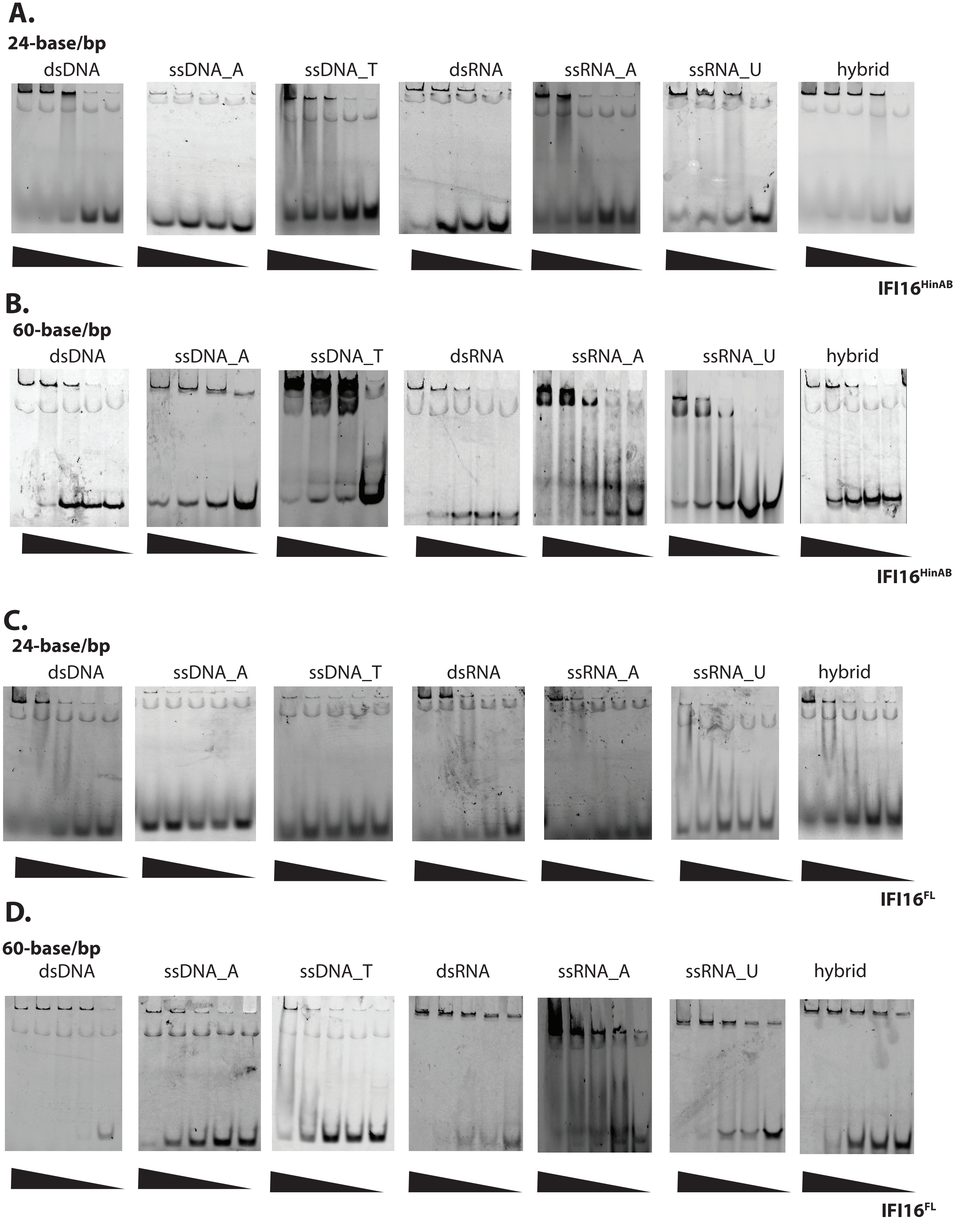

Supplementary Figure 3.

(**A**) EMSAs of IFI16^{HinAB} (1, 0.5, 0.25, 0.125, and 0 μM for dsDNA and ssRNA_A; 2, 1, 0.5, and 0 μM for the rest) binding various 24-bp/base FAM-labeled nucleic acids (20 nM).

(**B**) EMSAs of IFI16^{HinAB} (0.3, 0.1, 0.033, 0.011, and 0 μM for dsDNA, ssRNA_A/U, dsRNA, and the hybrid; 2, 1, 0.5, and 0 μM for ssDNA_A/T) binding various 60-bp/base FAM-labeled nucleic acids (20 nM).

(**C**) EMSAs of IFI16^{FL} (0.5, 0.25, 0.125, 0.0625, and 0 μM) binding various 24-bp/base FAM-labeled nucleic acids (20 nM).

(**D**) EMSAs of IFI16^{FL} (0.2, 0.1, 0.05, 0.025, and 0 μM) binding various 60-bp/base FAM-labeled nucleic acids (20 nM).

Supplementary Figure 4

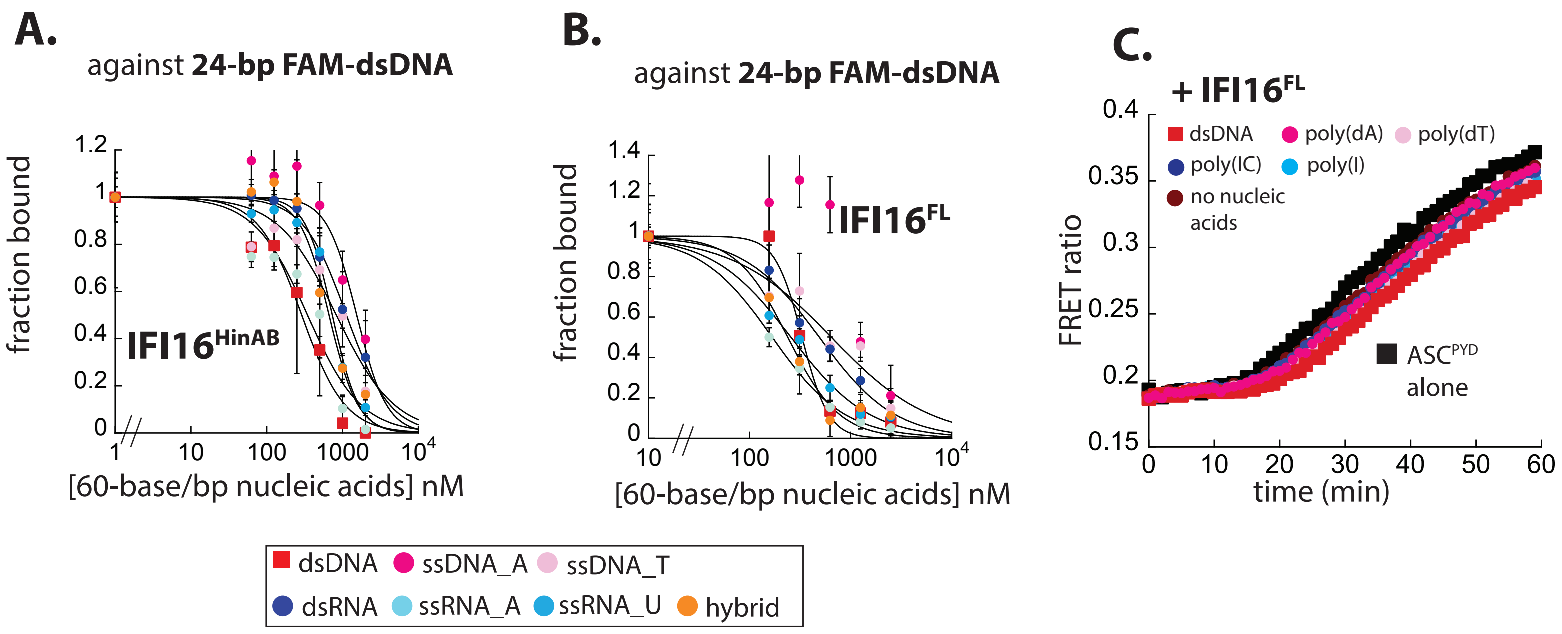

Supplementary Figure 4.

(**A-B**) Competition binding assay using 24-bp FAM-dsDNA (6 nM) and **IFI16^{HinAB}** ((**A**), 1 μM) and **IFI16^{FL}** ((**B**), 500 nM) vs. various 60-base/bp unlabeled nucleic acids; the lines are fits to the competition binding equation. Fraction bound was calculated based on the changes in FA of 24-bp FAM-dsDNA. Shown are averages of three experiments.

(**C**) The time-dependent increase in FRET emission ratio of donor- and acceptor-labeled ASC^{PYD} (2.5 μM) was monitored in the presence of preassembled IFI16^{FL} (600 nM) on 10 ng/μl of indicated nucleic acids/mimics (preincubated for 30 min).

Supplementary Figure 5

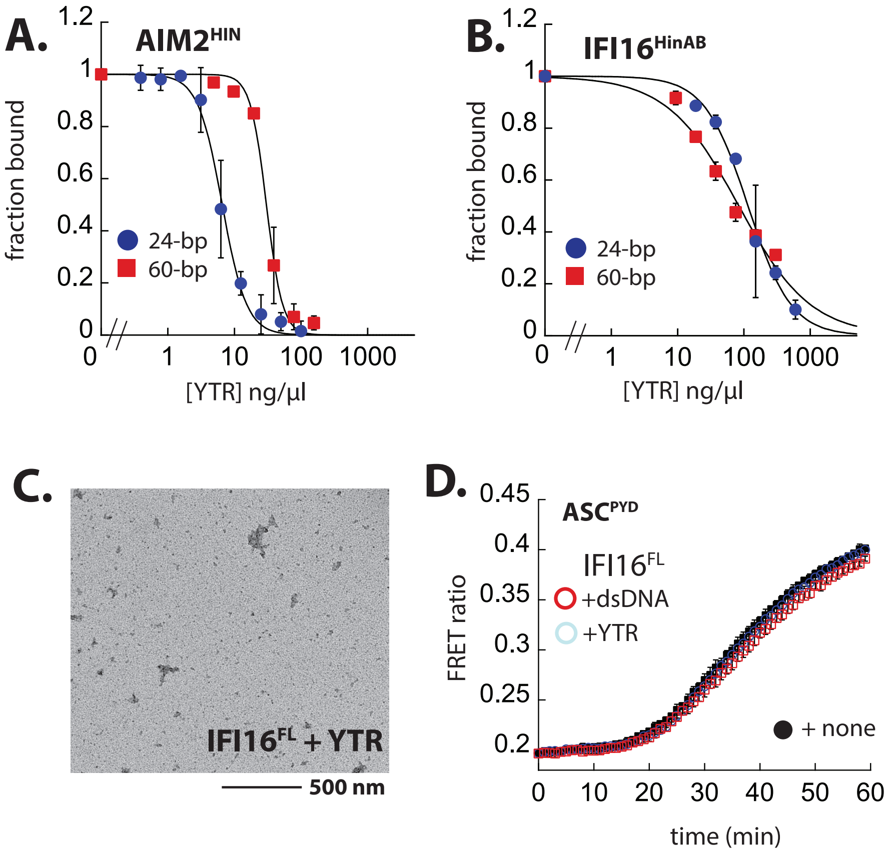

Supplementary Figure 5.

(**A-B**) Competition binding assays using 24- or 60-bp FAM-dsDNA (6 nM) and AIM2^{Hin} (625 nM and 400 nM, respectively, **A**), and IFI16^{HinAB} (1 µM and 400 nM, respectively, **B**) against yeast total RNA extract (YTR). Shown are averages of three experiments.

(**C**) A nsEM image of IFI16^{FL} (300 nM) in the presence of 600 ng/µl YTR did not produce any visible filaments.

(**D**) The time-dependent increase in FRET emission ratio of donor- and acceptor-labeled ASC^{PYD} (2.5 µM) was monitored in the presence of preassembled IFI16^{FL} (600 nM) on 10 ng/µl of 150-bp dsDNA and 600 ng/µl YTR (preincubated for 30 min). Shown are averages of three experiments with error bars (standard deviation).

Supplementary Tables

(**1**) Nucleic acid sequences used in the current study. Subscripts indicate the number of repeats of the given sequence. Only the sense strand is shown for double-stranded variants, and we used RNA as the anti-sense strand for the DNA:RNA hybrid.

**24-base/bp nucleic acids**	***Sequence***
dsDNA	TAAGACACGATGCGATAAAATCTG
ssDNA_A	$(AAAG)_6$
ssDNA_T	$(TTTC)_6$
dsRNA	r(UAAGACACGAUGCGAUAAAAUCUG)
ssRNA_U	$r(UUUC)_6$
ssRNA_A	$r(AAAG)_6$
DNA:RNA hybrid	TAAGACACGATGCGATAAAATCTG
**60-base/bp nucleic acids**	
dsDNA	TAAGACACGATGCGATAAAATCTGTTTGTA AAATTTATTAAGGGTACAAATTGCCCTAGC
ssDNA_A	$(AAAG)_{15}$
ssDNA_T	$(TTTC)_{15}$
dsRNA	r(UAAGACACGAUGCGAUAAAAUCUGUUUGUA AAAUUUAUUAAGGGUACAAAUUGCCCUAGC)
ssRNA_U	$r(UUUC)_{15}$
ssRNA_A	$r(AAAG)_{15}$
DNA:RNA hybrid	TAAGACACGATGCGATAAAATCTGTTTGTA AAATTTATTAAGGGTACAAATTGCCCTAGC

(2) Table summarizing the binding affinity of **AIM2^{HIN}** toward various FAM-labeled nucleic acids (6 nM). n = 3, ± SD.

**24-base/bp nucleic acids**	**K_D (μM)**	**Hill constant**
dsDNA	0.9 ± 0.2	2.4 ± 0.2
ssDNA_A	5.7 ± 0.7	1.1 ± 0.1
ssDNA_T	1.2 ± 0.2	2.0 ± 0.3
dsRNA	1.2 ± 0.2	2.5 ± 0.2
ssRNA_U	0.21 ± 0.06	2.6 ± 0.3
ssRNA_A	1.0 ± 0.1	1.7 ± 0.2
DNA:RNA hybrid	0.9 ± 0.1	2.2 ± 0.6
**60-base/bp nucleic acids**	**K_D (μM)**	**Hill constant**
dsDNA	0.6 ± 0.1	2.2 ± 0.1
ssDNA_A	2.7 ± 0.1	2.1 ± 0.1
ssDNA_T	0.4 ± 0.1	1.9 ± 0.2
dsRNA	1.1 ± 0.2	1.8 ± 0.2
ssRNA_U	0.3 ± 0.1	1.3 ± 0.2
ssRNA_A	1.4 ± 0.2	1.1 ± 0.1
DNA:RNA hybrid	0.8 ± 0.1	2.3 ± 0.2

(3) Table summarizing the binding affinity of **AIM2^{FL}** toward various FAM-labeled nucleic acids (3 nM). n = 3, ± SD. n.d.= not determined. * an upper-limit estimate as it is too close to the concentration (the detection limit of the instrument) of FAM-dsDNA. n.d.: not determined.

**24-base/bp nucleic acids**	**K_D (μM)**	**Hill constant**
dsDNA	0.08 ± 0.02	2.1 ± 0.4
ssDNA_A	> 0.5	n.d.
ssDNA_T	> 0.5	n.d.
dsRNA	> 0.5	n.d.
ssRNA_U	> 0.5	n.d.
ssRNA_A	> 0.5	n.d.
DNA:RNA hybrid	> 0.5	n.d.
**60-base/bp nucleic acids**	**K_D (μM)**	**Hill constant**
dsDNA	< 0.003*	n.d.
ssDNA_A	0.15 ± 0.03	1.2 ± 0.2
ssDNA_T	0.32 ± 0.08	0.8 ± 0.1
dsRNA	0.05 ± 0.01	0.7 ± 0.1
ssRNA_U	0.08 ± 0.02	0.8 ± 0.1
ssRNA_A	0.4 ± 0.1	2.1 ± 0.3
DNA:RNA hybrid	0.04 ± 0.01	2.1 ± 0.3

(4) Table summarizing the IC_{50} values of 60-base/bp nucleic acids against FAM-labeled dsDNA (6 nM) for **$AIM2^{HIN}$** (625 and 400 nM for 24- and 60-bp FAM-dsDNA, respectively). IC_{50}s were determined by fitting the average data points from three experiments shown in Figure 1. n.c. : no significant competition.

**Against 24-bp FAM-dsDNA**	**IC_{50} (nM)**	**Fitting error**
dsDNA	19.7	1.9
ssDNA_A	134.5	16.6
ssDNA_T	112.9	7.3
dsRNA	92.9	2.8
ssRNA_U	218.8	33.5
ssRNA_A	132.8	0.4
DNA:RNA hybrid	100.0	9.0
**Against 60-bp FAM-dsDNA**	**IC_{50} (nM)**	
dsDNA	267.7	8.4
ssDNA_A	n.c.	-
ssDNA_T	1000.0	259.0
dsRNA	281.2	53.1
ssRNA_U	740.7	70.3
ssRNA_A	1228.7	483.3
DNA:RNA hybrid	350.1	44

(5) Table summarizing the IC_{50} values of 60-base/bp nucleic acids against FAM-labeled dsDNA (6 nM) for **$AIM2^{FL}$** (100 and 60 nM for 24- and 60-bp FAM-dsDNA, respectively). IC_{50}s were determined by fitting the average data points from three experiments shown in Figure 1. n.c. : no significant competition.

**Against 24-bp FAM-dsDNA**	**IC_{50} (nM)**	**Fitting error**
dsDNA	11.8	0.3
ssDNA_A	58.9	6.7
ssDNA_T	165	7.3
dsRNA	69.7	6.7
ssRNA_U	273.0	171.0
ssRNA_A	46.1	2.7
DNA:RNA hybrid	19.6	0.44
**Against 60-bp FAM-dsDNA**	**IC_{50} (nM)**	
dsDNA	141.8	8.5
ssDNA_A	n.c.	-
ssDNA_T	n.c.	-
dsRNA	n.c.	-
ssRNA_U	n.c.	-
ssRNA_A	1017.0	129.7
DNA:RNA hybrid	n.c.	-

(6) Table summarizing apparent assembly rates (k_{assm}) of **AIM2^{FL}** oligomers on various nucleic acids. n = 3, ± SD. k_{assm}s were obtained by fitting the data with a single-exponential growth equation. Corresponding half-times ($t_{1/2}$s) were obtained by ln(2)/k_{assm}. The apparently faster k_{assm}s for 60-base/bp nucleic acids are caused by the higher protein concentration. n.d.= not determined.

**AIM2^{FL} (75 nM)**		
**60-base/bp nucleic acids**	**k_{assm} (min^{-1})**	**$t_{1/2}$ (min)**
dsDNA	0.19 ± 0.06	3.5
ssDNA_A	0.08 ± 0.03	8.2
ssDNA_T	0.07 ± 0.01	10.5
dsRNA	0.07 ± 0.02	10.5
ssRNA_U	0.04 ± 0.01	16.4
ssRNA_A	0.07 ± 0.02	9.6
DNA:RNA hybrid	0.10 ± 0.03	7.2
**AIM2^{FL} (9 nM)**		
**"longer" nucleic acids**	**k_{assm} (min^{-1})**	**$t_{1/2}$ (min)**
dsDNA (300-bp)	0.21 ± 0.03	3.3
poly(dT)	0.06 ± 0.01	11.7
poly(dA)	n.d.	n.d.
poly(IC)	0.04 ± 0.01	17.9
poly(I)	0.68 ± 0.04	1.0

(7) Table summarizing the binding affinity of **IFI16^{HinAB}** toward various FAM-labeled nucleic acids (3 nM). n = 3, ± SD. n.d.= not determined.

**IFI16^{HinAB}**		
**60-base/bp nucleic acids**	**K_D (μM)**	**Hill constant**
dsDNA	0.02 ± 0.01	0.9 ± 0.2
ssDNA_A	0.5 ± 0.2	1.5 ± 0.2
ssDNA_T	0.07 ± 0.02	1.1 ± 0.1
dsRNA	0.03 ± 0.01	1.2 ± 0.2
ssRNA_U	0.03 ± 0.01	1.1 ± 0.2
ssRNA_A	0.06 ± 0.02	1.2 ± 0.1
DNA:RNA hybrid	0.06 ± 0.01	0.9 ± 0.1

(8) Table summarizing the binding affinity of **IFI16^{FL}** toward various FAM-labeled nucleic acids (3 nM). *n* = 3, ± SD. n.d.= not determined. *: an upper-limit estimate as it is too close to the concentration (the detection limit of the instrument) of FAM-dsDNA.

**IFI16^{FL}**		
**60-base/bp nucleic acids**	**K_D (μM)**	**Hill constant**
dsDNA	0.003*	1.8 ± 0.2
ssDNA_A	0.15 ± 0.02	1.5 ± 0.2
ssDNA_T	0.022 ± 0.004	0.7 ± 0.2
dsRNA	0.085 ± 0.007	0.7 ± 0.2
ssRNA_U	0.078 ± 0.011	0.6 ± 0.1
ssRNA_A	0.008 ± 0.002	0.6 ± 0.1
DNA:RNA hybrid	0.035 ± 0.015	0.6 ± 0.1

(9) Table summarizing the IC_{50} values of 60-base/bp nucleic acids against FAM-labeled dsDNA (6 nM) for **IFI16^{HinAB}** (1000 and 400 nM for 24- and 60-bp FAM-dsDNA, respectively) IC_{50}s were determined by fitting the average data points from three experiments shown in Figure 4 and Supplementary Figure 3. n.c. : no significant competition.

**Against 24-bp FAM-dsDNA**	**IC_{50} (nM)**	**Fitting error**
dsDNA	288.9	37.3
ssDNA_A	1538.3	255.7
ssDNA_T	827.7	154.8
dsRNA	1126.2	61.7
ssRNA_U	733.1	48.5
ssRNA_A	344.8	70.6
DNA:RNA hybrid	663.2	64.6
**Against 60-bp FAM-dsDNA**	**IC_{50} (nM)**	**Fitting error**
dsDNA	257.8	24.9
ssDNA_A	3013.3	1242.4
ssDNA_T	1387.5	136.6
dsRNA	1430.0	365.9
ssRNA_U	n.c.	n.c.
ssRNA_A	1966.6	90.0
DNA:RNA hybrid	623.4	106.4

(10) Table summarizing the IC_{50} values of 60-base/bp nucleic acids against FAM-labeled dsDNA (6 nM) for **$IFI16^{FL}$** (500 and 100 nM for 24- and 60-bp FAM-dsDNA, respectively). IC_{50}s were determined by fitting the average data points from duplicate experiments shown in Figure 4 and Supplementary Figure 3. n.c. : no significant competition.

**Against 24-bp FAM-dsDNA**	**IC_{50} (nM)**	**Fitting error**
dsDNA	330.8	29.8
ssDNA_A	1416.8	318.2
ssDNA_T	644.0	133.2
dsRNA	489.8	40.3
ssRNA_U	305	48.5
ssRNA_A	167.6	11.6
DNA:RNA hybrid	238.8	31.9
**Against 60-bp FAM-dsDNA**	**IC_{50} (nM)**	**Fitting error**
dsDNA	627.2	132.3
ssDNA_A	n.c.	-
ssDNA_T	n.c.	-
dsRNA	1072.9	132.3
ssRNA_U	1120.2	75.5
ssRNA_A	165.6	18.0
DNA:RNA hybrid	975.1	48.1

(11) Table summarizing apparent assembly rates (k_{assm}) of IFI16^{FL} oligomers on various nucleic acids. $n = 3$, ± SD. For 60-base/bp nucleic acids, k_{assm}s were obtained by fitting the data with a single-exponential growth equation. Half-times ($t_{1/2}$s) were obtained by $\ln(2)/k_{assm}$. For the longer nucleic acids, we used a variant of the Hill equation to estimate the half-time for each experiment (ref. 37), which was then converted to k_{assm}.

**IFI16^{FL} (300 nM)**		
**60-base/bp nucleic acids**	**k_{assm} (min^{-1})**	**$t_{1/2}$ (min)**
dsDNA	0.26 ± 0.05	2.7
ssDNA_A	0.17 ± 0.04	4.1
ssDNA_T	n.d.	n.d.
dsRNA	0.014 ± 0.002	47.9
ssRNA_U	0.025 ± 0.004	28.1
ssRNA_A	0.037 ± 0.001	9.6
DNA:RNA hybrid	0.09 ± 0.02	7.7
**IFI16^{FL} (60 nM)**		
**“longer” nucleic acids**	**k_{assm} (min^{-1})**	**$t_{1/2}$ (min)**
dsDNA (150-bps)	0.046	14.8 ± 2.9
poly(dT)	0.013	53.9 ± 7.1
poly(dA)	n.d.	n.d.
poly(IC)	0.026	26.7 ± 3.3
poly(I)	0.013	54.7 ± 5.3

(12) Table summarizing the IC_{50} values of YTR against FAM-labeled dsDNA (6 nM) for **ALRs.** The same amounts of ALRs were used as in other competition experiments. IC_{50}s were determined by fitting the average data points from three experiments shown in Figure 5 and Supplementary Figure 5. IC_{50} values against 60-bp in mass-concentrations are shown in italics for comparison (converted from molar concentrations indicated in Table 4, 5, 9, and 10).

	60-bp dsDNA	**YTR**	**YTR**
**AIM2^{HIN}**	***IC_{50} (ng/µl)***	**IC_{50} (ng/µl)**	**Fitting error**
24-bp FAM-dsDNA	*0.7*	6.6	0.3
60-bp FAM-dsDNA	*9.9*	30.2	1.3
**AIM2^{FL}**			
24-bp FAM-dsDNA	*0.4*	7.2	0.3
60-bp FAM-dsDNA	*5.2*	167.7	17.3
**IFI16^{HinAB}**		**IC_{50} (ng/µl)**	**Fitting error**
24-bp FAM-dsDNA	*10.7*	115.4	7.1
60-bp FAM-dsDNA	*9.5*	83.3	8.9
**IFI16^{FL}**			
24-bp FAM-dsDNA	*12.2*	30.8	4.1
60-bp FAM-dsDNA	*23.2*	31.8	0.6
